# Association of *NOTCH4* and *CYP2E1* Genetic Variants With Schizophrenia in the Bangladeshi Population: A Case‐Control Study

**DOI:** 10.1002/hsr2.70262

**Published:** 2024-12-17

**Authors:** Md. Shalahuddin Millat, Joysree Roy, Md. Atikur Rahman, Md. Abdul Aziz, Safiqul Islam, Md. Mazharul Islam Chowdhury, Md Abdul Barek, Md. Saddam Hussain, Mohammad Sarowar Uddin, Shafayet Ahmed Siddiqui, Mohammad Safiqul Islam

**Affiliations:** ^1^ Department of Pharmacy Noakhali Science and Technology University Sonapur Noakhali Bangladesh; ^2^ Laboratory of Pharmacogenomics and Molecular Biology, Department of Pharmacy Noakhali Science and Technology University Sonapur Noakhali Bangladesh; ^3^ Department of Pharmaceutical Sciences North South University Dhaka Bangladesh

**Keywords:** *CYP2E1*, genotyping, *NOTCH4*, PCR–RFLP, schizophrenia

## Abstract

**Background and Aims:**

Schizophrenia (SCZ) is among the most persistent and devastating psychological problems. Different genetic polymorphisms are responsible for the predisposition of SCZ, and we screened *NOTCH4* (rs2071287, rs204993) and *CYP2E1* (rs2070673) polymorphisms in this study to find the connection with SCZ development.

**Methods:**

We investigated a total of 420 samples (210 patients and 210 controls) and used the PCR–RFLP technique to genotype all SNPs. For statistical analyses, SPSS (version 25.0) was applied.

**Results:**

In the case of *NOTCH4* rs2071287, no evidence of a link was found in any genetic models, whereas *NOTCH4* rs204993 and *CYP2E1* rs2070673 showed a significant linkage in four genetic models with SCZ risk (for *NOTCH4* rs204993, additive model 2: OR = 3.39, CI = 1.84–6.23, *p* = 0.0001; dominant: OR = 1.84, CI = 1.22–2.76, *p* = 0.0032; recessive: OR = 2.67, CI = 1.53–4.64, *p* = 0.0005; allelic: OR = 1.75, CI = 1.32–2.30, *p* = 0.0001 and for *CYP2E1* rs2070673, additive model 2: OR = 0.39, CI = 0.22–0.69, *p* = 0.0013; recessive: OR = 0.45, CI = 0.29–0.68, *p* = 0.0002; overdominant: OR = 1.49, CI = 1.02–2.19, *p* = 0.0408; allelic: OR = 0.61, CI = 0.46–0.80, *p* = 0.0004).

**Conclusions:**

The findings of our study suggest that the polymorphisms *NOTCH4* rs204993 and *CYP2E1* rs2070673 in the Bangladeshi ethnicity are connected to the risk of SCZ.

## 1 Introduction

Schizophrenia (SCZ) is a prolonged polygenic condition marked by both positive (e.g., illusions, infatuation, and mental abnormalities) and negative signs and symptoms (e.g., cognitive impairment, aversion, and social withdrawal) [1, 2]. Globally, it is regarded as one of the most pressing issues related to public health and making it the ninth most frequently occurring human mental disorder [3]. Although the prevalence of SCZ in childhood and the later half of life (over 45 years) is not too high, the apprehension of childhood‐onset SCZ is progressively increasing [4, 5]. The ratio of developing schizophrenic disorder between women and men is 1:1.4, and men seem to be diagnosed early in life than women [6]. SCZ mainly affects men in the age range of 18 and 25 years, while the age of onset is 25–35 years for women, with a higher occurrence during menopause [7].

A wide variety of psychotic complications of schizophrenic disorder make this the most disabling illness, which leads the patients' personal, familial, and social life into a complicated state. The pathophysiology of SCZ remains indistinct despite performing hundreds of studies on this disorder [8]. However, it is now widely accepted that SCZ is caused by various elements, for example, genetic, environmental, or psychological factors. Evidence suggests that genetic variables like family background, twin babies, as well as gene polymorphism play a pivotal role in the pathogenesis of this condition [9, 10]. Despite the fact that applicant gene strategies and genome‐wide epidemiological studies for SCZ have yielded a lot of evidence, only a small number of genes have been thoroughly investigated across ethnic groups [11, 12]. As a result, research into the specific causes and mechanisms behind the link of genetic variants with the susceptibility of SCZ in diverse ethnicity or population is essential.

Bangladesh is a highly populated country, and a large portion of its population seems to suffer from this major psychiatric ailment [13]. As per a survey performed in the outpatient clinics of the National Institute of Mental Health (NIMH) in Dhaka, Bangladesh, more than 35% of outpatients had SCZ and SCZ‐like psychological symptoms, 11.19% had a depressive episode, 8.95% had manic depressive disorder, and 16.14% had anxiety disorders [14]. Another study in NIMH reported that among all SCZ patients, 25% healed through appropriate treatment, 25%–35% led regular lives by taking medication on a regular basis, 10%–15% never recovered from the illness, and 10%–15% died themselves or settled suicidal trend [15]. Remarkably, the most frequent psychiatric illness types are seen in rural and urban Bangladeshi residents [16].

On chromosome 6 (6p21.3), *NOTCH4* (neurogenic locus notch homolog protein 4 gene) encodes a member of the *NOTCH* class, a critical neurodevelopment‐related gene. The differentiation, proliferation, and death of brain cells are all controlled by *NOTCH* through the process of creating and morphogenesis of organs [17]. The maturity signaling of neural cells from progenitor cells is also governed by this gene [18, 19, 20]. Not only does the gene play a crucial part in neurodevelopment, but the transcripts of the *NOTCH4* gene make it a viable candidate for the progression of neurological illnesses like SCZ [21, 22]. *NOTCH4* polymorphisms (e.g., rs3131296, rs3132935, rs3809842) have been explained by some prior investigations (e.g., rs3132935) [23, 24, 25]. The rs2071287 SNP of the *NOTCH4* gene has been found to be the most statistically significant marker for SCZ in the Japanese population [26]. The rs204993 polymorphism in *NOTCH4* was reported to be correlated with the susceptibility of SCZ in a family‐based investigation of 218 Taiwanese families that are made up of only one person [27]. According to these findings, SCZ risk may be linked with rs2071287 and rs204993. An important representative of the cytochrome P450 enzyme superfamily, the *CYP2E1* gene is responsible for the metabolic activities and biotransformation of a wide range of low molecular weight drugs. Interindividual heterogeneity in medication response, drug‐drug interactions, and various psychiatric illnesses are all linked to *CYP2E1* genetic polymorphisms [28, 29]. *CYP2E1* contributes to hepatic pathologies that result from alcoholic and nonalcoholic steatohepatitis [28], renal impairment [30], and so forth. A cluster analysis of transcriptional alterations in the Chinese Han population showed that *CYP2E1* gene variants might be related to SCZ [29]. These outcomes pose the probability of the association between rs2070673 polymorphism with SCZ risk.

Geographical and cultural differences in genetic makeup significantly impact the frequency and severity of disease instances around the world. As a result, people of various ethnicities and backgrounds get varied results, sometimes even within the same ethnic group [31, 32]. Therefore, *CYP2E1* rs2070673 and *NOTCH4* rs2071287 and rs204993 have not been studied for genetic connections in the Bangladeshi population. As a result, the current study sought to determine whether gene polymorphisms associated with these SCZ variants existed.

## 2 Methods

### 2.1 Selection of Subjects

The experimental protocol (NSTU/REG/AC/2022/4627 (09)) was checked and approved by the institutional review committee of the Noakhali Science and Technology University, Noakhali‐3814, Bangladesh. All patients and controls were asked to sign a consent form after being fully briefed on the study's aims and objectives.

All of the patients in this study were obtained from the NIMH, Dhaka‐1207, Bangladesh. This case‐control analysis included 210 schizophrenic subjects and 210 healthy participants who were matched by age and gender to the cases from a separate part of Bangladesh. It was determined that at least two senior psychiatrists had to concur on a diagnosis after evaluating the patient's personal history, family history report, and clinical history to meet the diagnostic requirements of the Diagnostic and Statistical Manual of Mental Disorders–5th Edition (DSM‐V). Demographic variables such as age, sex, and educational level of the healthy controls were assimilated with the patients and also verified not to have any mental distress. This study did not include healthy individuals under the age of 18 who were unable to supply the required data. The present research omitted participants with cancer or other illnesses like kidney, liver, or lung disease. This analysis was completed in accordance with the updated Helsinki Declaration and its subsequent amendments [33]. After the collection of whole blood samples, the specimens were placed in plastic tubes equipped with EDTA (Ethylenediaminetetraacetic acid) and kept at −80°C before they were ready for DNA extraction. We extracted the DNA using a Favorgen (Taiwan) DNA Extraction Mini Kit and assessed the concentration with a spectrophotometer (Genova Nano, Jenway). The quality of the genomic DNA samples was confirmed, setting the OD 260/OD 280 ratio between 1.7 and 1.9 and the average concentration between 50 and 70 µg/mL.

### 2.2 Genotyping Method

For genotyping all three variants (rs2071287, rs204993, and rs2070673), the polymerase chain reaction‐restriction fragment length polymorphism (PCR–RFLP) genotyping method was implemented. For conducting the PCR technique, a PCR working mix was prepared by adding both designed forward and reverse primers at appropriate concentrations in EmeraldAmp GT PCR Master Mix (2×). Then PCR tube was taken to transfer the 20 µL of working mix followed by 1 µL of extracted DNA to make the total volume of 21 µL. After that, the PCR was performed at validated conditions to amplify the targeted gene. The primers were designed using primer blast. The PCR products for rs2071287, rs204993, and rs2070673 were 306, 243, and 218 bytes, respectively, and were examined using agarose (1% w/v) gel electrophoresis. The restriction enzymes were then used to digest the PCR results, as well as the digested samples were visualized on a 2% (w/v) agarose gel. To determine genotypes, 20% of the total heterozygotes and all mutated homozygotes were sorted again [9]. Table 1 lists the primers used in this work with polymorphism characteristics generated from HaploReg, and Table 2 shows the fragmented patterns alongside genotypes for all alleles.

**Table 1:** SNPs characteristics with primers sequences.

Genes	SNPs	Position (role)	Predicted function	Minor allele	MAF	Sequences (5′–3′)
*NOTCH4*	rs2071287	Chr 6:32202656 (Intron)	Promoter/enhancer histone mark, motifs changed, GRASP QTL hits, Selected eQTL hits	A	0.37	FP: 5′‐CTAGGACCCTCTGCACCGTC‐3′ RP: 5′‐GCCTATACAATGGCAGCTGC‐3′
	rs204993	Chr6: 32187804 (Intron)	Enhancer histone mark, GRASP QTL hits, Selected eQTL hits	C	0.34	FP: 5′‐ATGACAATGGTGACTCTGGGG‐3′ FP: 5′‐AGGTATTATGGAGGTTGCGGG‐3′
*CYP2E1*	rs2070673	Chr10:133527063 (Intron)	Promoter/enhancer histone mark, motifs changed, Selected eQTL hits	T	0.63	FP‐5′‐CTGGAGTTCCCCGTTGTCTA‐3′ RP:5′‐CCATCGTTTCAAAGGCTGAT‐3′

**Table 2:** Fragmentation pattern of alleles with polymorphism.

SNPs	Restriction enzyme	Digestion condition	Genotype	PCR product size (bp)	Fragmentation (bp)
*NOTCH4* rs2071287	Bcc*I*	Incubation at 37°C for overnight	GG AG AA	306	N: 34,121,151 H: 34,121,151,155 M: 151,155
*NOTCH4* rs204993	BsrB*I*	Incubation at 37°C for overnight	TT CT CC	243	N: 243 H: 68,175,243 M: 68,175
*CYP2E1* rs2070673	BspM*I*	Incubation at 37°C for overnight	AA AT TT	218	N: 218 H: 26,192,218 M: 26,192

Abbreviations: H, heterozygous; M, mutant homozygous; N, normal homozygous.

### 2.3 False Positive Report Probability (FPRP) and Bayesian False Discovery Probability (BFDP)

To evaluate the significance of the results, the FPRP was determined. To find an odds ratio (OR) of 1.5 associated with cancer risk in the study, we fixed the threshold for FPRP at 0.2 and defined prior probabilities of 0.25, 0.1, 0.01, 0.001, and 0.0001. Only findings with FPRP values less than 0.50 were deemed noteworthy. To evaluate the validity of statistically significant correlations, the BFDP was calculated using an Excel computation spreadsheet. BFDP levels below 0.8 were regarded as noteworthy.

### 2.4 Genotype‐Based Gene Expression Analysis

The UALCAN database (http://ualcan.path.uab.edu/) was used for in silico genotype‐based gene expression, and data were retrieved from the genotype‐tissue expression (GTEx) portal.

### 2.5 Statistical Analysis

Using the chi‐squared ( *χ* ^2^) contingency test, we were able to determine the Hardy–Weinberg equilibrium (HWE) and the genotype data discrepancy of the patients from their control group. The MedCalc software tool enumerated the OR with a corresponding 95% confidence interval (CI). To effectively carry out the other statistical analyses, we used SPSS (version 25.0). For the purposes of all analyses, a *p* value of ≤ 0.05 was fixed as statistically significant.

## 3 Results

### 3.1 Sample Characteristics and Genotype Data Distributions

In this study, schizophrenic subjects and controls comprised an average age of 27.84 ± 5.27 and 25.58 ± 5.32 years, respectively. The percentages of males and females in the controls were 85.71% and 14.29%, and that for patients were 85.24% and 14.76%. All variants, including rs2071287 and rs204993 of *NOTCH4* and rs2070673 of *CYP2E1*, showed no deviation (rs2071287: *p* = 0.609 and 0.714; rs204993: *p* = 0.713 and 0.354; rs2070673: *p* = 0.166 and 0.549) from HWE for both case and control groups, respectively. The frequency (%) of all alleles among SCZ patients and healthy controls and HWE of all variants are given in Table 3.

**Table 3:** Genotype data distributions of *NOTCH4* rs2071287, *NOTCH4* rs204993, and *CYP2E1* rs2070673.

SNPs	Schizophrenic patients (*n* = 210) (%)		HWE		Healthy subjects (*n* = 210) (%)	HWE	
			*χ* ^2^	*p* value		*χ* ^2^	*p* value
*NOTCH4* rs2071287							
GG AG AA G allele A allele	81 (38.57) 96 (45.71) 33 (15.71) 258 (61.43) 162 (38.57)		0.26	0.609	83 (39.52) 100 (47.62) 27 (12.86) 266 (63.33) 154 (36.67)	0.134	0.714
*NOTCH4* rs204993							
TT CT CC T allele C allele	60 (28.57) 102 (48.57) 48 (22.86) 222 (52.85) 198 (47.14)		0.135	0.713	89 (42.38) 100 (47.61) 21 (10.00) 278 (66.19) 142 (33.81)	0.86	0.354
*CYP2E1* rs2070673							
AA AT TT A Allele T Allele	46 (21.90) 115 (54.76) 49 (23.33) 207 (49.29) 213 (50.71)		1.91	0.166	31 (14.76) 94 (44.76) 85 (40.48) 156 (37.14) 264 (62.86)	0.36	0.549

Abbreviation: HWE, Hardy–Weinberg equilibrium.

### 3.2 Association of *NOTCH4* (rs2071287 and rs204993) and *CYP2E1* (rs2070673) Variants With SCZ

Table 4 shows the genotypic association of *NOTCH4* rs2071287, rs204993, and *CYP2E1* rs2070673 with SCZ risk in Bangladeshi populations. As is presented, this case‐control study found a higher percentage of heterozygous genotypes in all variants of both patients and healthy individuals. Though the frequency of the normal homozygous was higher than the frequency of the mutant homozygous in both cases and controls in the case of *NOTCH4* rs2071287and rs204993 polymorphisms; however, the mutant homozygous was more prevalent than the normal homozygous in both patients and healthy individuals with *CYP2E1* rs2070673 variants.

**Table 4:** Quantitative risk analysis of *NOTCH4* rs2071287, rs204993, and *CYP2E1* rs2070673 variants with SCZ risk in Bangladeshi populations.

SNPs	Models	Genotype/allele	Case (%)	Control (%)	OR	95% CI	*p* value
*NOTCH4* rs2071287		GG	81 (38.57)	83 (39.52)	1		
	Additive model 1 (AG vs. GG)	AG	96 (45.71)	100 (47.62)	0.98	0.65–1.49	0.9381
	Additive model 2 (AA vs. GG)	AA	33 (15.71)	27 (12.86)	1.25	0.69–2.27	0.4574
	Dominant model (AG + AA vs. GG)	GG	81 (38.57)	83 (39.52)	1		
		AG + AA	129 (61.43)	127 (60.48)	1.04	0.70–1.54	0.8415
	Recessive model (AA vs. GG + AG)	GG + AG	177 (84.29)	183 (87.14)	1		
		AA	33 (15.71)	27 (12.86)	1.26	0.73–2.19	0.4035
	Overdominant model (AG vs. GG + AA	GG + AA	114 (54.29)	110 (52.38)	1		
		AG	96 (45.71)	100 (47.62)	0.93	0.63–1.36	0.6956
	Allele (A vs. G)	G	258 (61.43)	266 (63.33)	1		
		A	162 (38.57)	154 (36.67)	1.08	0.82–1.43	0.5688
*NOTCH4* rs204993		TT	60 (28.57)	89 (42.38)	1		
	Additive model 1 (CT vs. TT)	CT	102 (48.57)	100 (47.61)	1.51	0.99–2.32	0.0580
	Additive model 2 (CC vs. TT)	CC	48 (22.86)	21 (10.00)	3.39	1.84–6.23	**0.0001**
	Dominant model (CT + CC vs. TT)	TT	60 (28.57)	89 (42.38)	1		
		CT + CC	150 (71.63)	121 (57.62)	1.84	1.22–2.76	**0.0032**
	Recessive model (CC vs. TT + CT)	TT + CT	162 (77.14)	189 (90.00)	1		
		CC	48 (22.86)	21 (10.00)	2.67	1.53–4.64	**0.0005**
	Overdominant model (CT vs. TT + CC	TT + CC	108 (51.43)	110 (52.38)	1		
		CT	102 (48.57)	100 (47.61)	1.04	0.71–1.52	0.8451
	Allele (C vs. T)	T	222 (52.85)	278 (66.19)	1		
		C	158 (47.16)	142 (33.81)	1.75	1.32–2.30	**0.0001**
*CYP2E1* rs2070673		AA	46 (21.90)	31 (14.76)	1		
	Additive model 1 (AT vs. AA)	AT	115 (54.76)	94 (44.76)	0.82	0.48–1.40	0.4760
	Additive model 2 (TT vs. AA)	TT	49 (23.33)	85 (40.48)	0.39	0.22–0.69	**0.0013**
	Dominant model (AT + TT vs. AA)	AA	46 (21.90)	31 (14.76)	1		
		AT + TT	164 (78.10)	179 (85.24)	0.62	0.37–1.02	0.0599
	Recessive model (TT vs. AA + AT)	AA + AT	161 (76.67)	125 (59.52)	1		
		TT	49 (23.33)	85 (40.48)	0.45	0.29–0.68	**0.0002**
	Overdominant model (AT vs. AA + TT	AA + TT	105 (45.24)	116 (55.24)	1		
		AT	115 (54.76)	94 (44.76)	1.49	1.02–2.19	**0.0408**
	Allele (T vs. A)	A	207 (49.29)	156 (37.14)	1		
		T	213 (50.71)	264 (62.86)	0.61	0.46–0.80	**0.0004**

*Note: p* < 0.05 was considered statistically significant. Bold values are indicate the statistically significant.

Abbreviation: OR, odds ratio.

Though both the stimulant and protective nature of *NOTCH4* rs2071287 variants in SCZ risk was found in different genetic inheritance models; however, no significant genotypic association was found in any genetic models, including additive model 1 (OR = 0.98, CI = 0.65–1.49, *p* = 0.9381), additive model 2 (OR = 1.25, CI = 0.69–2.27, *p* = 0.4574), dominant model (OR = 1.04, CI = 0.70–1.54, *p* = 0.8415), recessive model (OR = 1.26, CI = 0.73–2.19, *p* = 0.4035), overdominant model (OR = 0.93, CI = 0.63–1.36, *p* = 0.6956), and allelic model (OR = 1.08, CI = 0.82–1.43, *p* = 0.5688). Unlike the first variant discussed above, aside from the additive model 1 and overdominant model, all other genetic models showed a strong genotypic association of *NOTCH4* rs204993 polymorphisms with SCZ risk in Bangladeshi populations (additive model 1: OR = 1.51, CI = 0.99–2.32, *p* = 0.0580; additive model 2: OR = 3.39, Cl: 1.84–6.23, *p* = 0.0001; dominant model: OR = 1.84, CI = 1.22–2.76, *p* = 0.0032; recessive model: OR = 2.67, CI = 1.53–4.64, *p* = 0.0005; overdominant model: OR = 1.04, CI = 0.71–1.52, *p* = 0.8451; and allelic model: OR = 1.75, CI = 1.32–2.30, *p* = 0.0001).

In the case of *CYP2E1* rs2070673 (A/T) polymorphisms, all genetic inheritance models, except additive model 1 and the dominant model, demonstrated the strong association of this gene with SCZ risk in Bangladeshi populations. However, the genotypic association of this gene with increased SCZ risk was identified in only the overdominant model (OR = 1.49, CI = 1.02–2.19, *p* = 0.0408). Besides, the strong protective nature of this gene with SCZ risk was found in all other genetic models, including additive model 2 (OR = 0.39, CI = 0.22–0.69, *p* = 0.0013), recessive model (OR = 0.45, CI = 0.29–0.68, *p* = 0.0002), and allelic model (OR = 0.61, CI = 0.46–0.80, *p* = 0.0004). Though a protective nature was also observed in both additive model 1 and the dominant model, however, this association was not statistically significant (additive model 1: OR = 0.82, CI = 0.48–1.40, *p* = 0.4760; dominant model: OR = 0.62, CI = 0.37–1.02, *p* = 0.0599).

### 3.3 FPRP and BFDP Test

Table 5 displays the FPRP values of statistical power for significant findings with the *NOTCH4* rs2071287, *NOTCH4* rs204993, and *CYP2E1* rs2070673 polymorphisms. The FPRP analyses revealed that almost all *NOTCH4* rs204993 and *CYP2E1* rs2070673 polymorphisms models were noteworthy by FPRP estimation at the OR of 1.5 with the prior probability of 0.25 and 0.1, but noteworthy findings were obtained only for three models in each of *NOTCH4* rs204993 and *CYP2E1* rs2070673 polymorphisms by BFDP test at the OR of 1.5 with the prior probability of 0.01, 0.001.

**Table 5:** False‐positive report probability values for the association between *NOTCH4* rs2071287, *NOTCH4* rs204993, and *CYP2E1* rs2070673 and the risk of schizophrenia.

Genotype	Crude OR (95% CI)	*p*	Statistical power			Prior probability									BFDP prior probability		
						0.25		0.1		0.01		0.001		0.0001	0.01	0.001	0.00001
*NOTCH4* rs2071287																	
AG versus GG	0.98 (0.65–1.49)	0.9381	0.964			0.742		0.896		0.990		0.999		1.000	0.996	1.000	1.000
AA versus GG	1.25 (0.69–2.27)	0.4574	0.725			0.657		0.852		0.984		0.998		1.000	0.994	0.999	1.000
AG + AA versus GG	1.04 (0.70–1.54)	0.8415	0.966			0.724		0.887		0.989		0.999		1.000	0.997	1.000	1.000
AG versus GG + AA	0.93 (0.63–1.36)	0.6956	0.957			0.689		0.869		0.987		0.999		1.000	0.996	1.000	1.000
AA versus GG + AG	1.26 (0.73–2.19)	0.4035	0.732			0.628		0.835		0.982		0.998		1.000	0.994	0.999	1.000
A versus G	1.08 (0.82–1.43)	0.5688	0.989			0.642		0.843		0.983		0.998		1.000	0.997	1.000	1.000
*NOTCH4* rs204993																	
CT versus TT	1.51 (0.99–2.32)	0.0580	0.488			**0.269**		0.525		0.924		0.992		0.999	0.983	0.998	1.000
CC versus TT	3.39 (1.84‐ 6.23)	**0.0001**	0.004			**0.055**		**0.149**		0.659		0.951		0.995	**0.355**	0.848	0.998
CT + CC versus TT	1.84 (1.22–2.76)	**0.0032**	0.162			**0.056**		**0.151**		0.662		0.952		0.995	0.862	0.984	1.000
CT versus TT + CC	1.04 (0.71 − 1.52)	0.8451	0.971			0.722		0.886		0.988		0.999		1.000	0.997	1.000	1.000
CC versus TT + CT	2.67 (1.53 − 4.64)	**0.0005**	0.020			**0.068**		**0.179**		0.706		0.960		0.996	**0.635**	0.946	0.999
C versus T	1.75 (1.32 −2.30)	**0.0001**	0.134			**0.001**		**0.004**		**0.042**		**0.308**		0.817	**0.172**	**0.678**	0.995
*CYP2E1* rs2070673																	
AT versus AA	0.82 (0.48 −1.40)	0.4760	0.776			0.644		0.844		0.983		0.998		1.000	0.995	0.999	1.000
TT versus AA	0.39 (0.22 − 0.69)	**0.0013**	0.033			**0.100**		**0.251**		0.786		0.974		0.997	**0.777**	0.972	1.000
AT + TT vs AA	0.62 (0.37 − 1.02)	0.0599	0.388			**0.317**		0.581		0.939		0.994		0.999	0.982	0.998	1.000
AT versus AA + TT	1.49 (1.02 −2.19)	**0.0408**	0.514			**0.199**		**0.426**		0.891		0.988		0.999	0.980	0.998	1.000
TT versus AA + AT	0.45 (0.29 − 0.68)	**0.0002**	0.031			**0.014**		**0.042**		**0.324**		0.829		0.980	**0.342**	0.840	0.998
T versus A	0.61 (0.46 −0.80)	**0.0004**	0.260			**0.004**		**0.012**		**0.118**		0.575		0.931	**0.502**	0.910	0.999

Bold values are indicate the statistically significant.

### 3.4 Genotype‐Based Gene Expression Analysis

**Figure 1:**
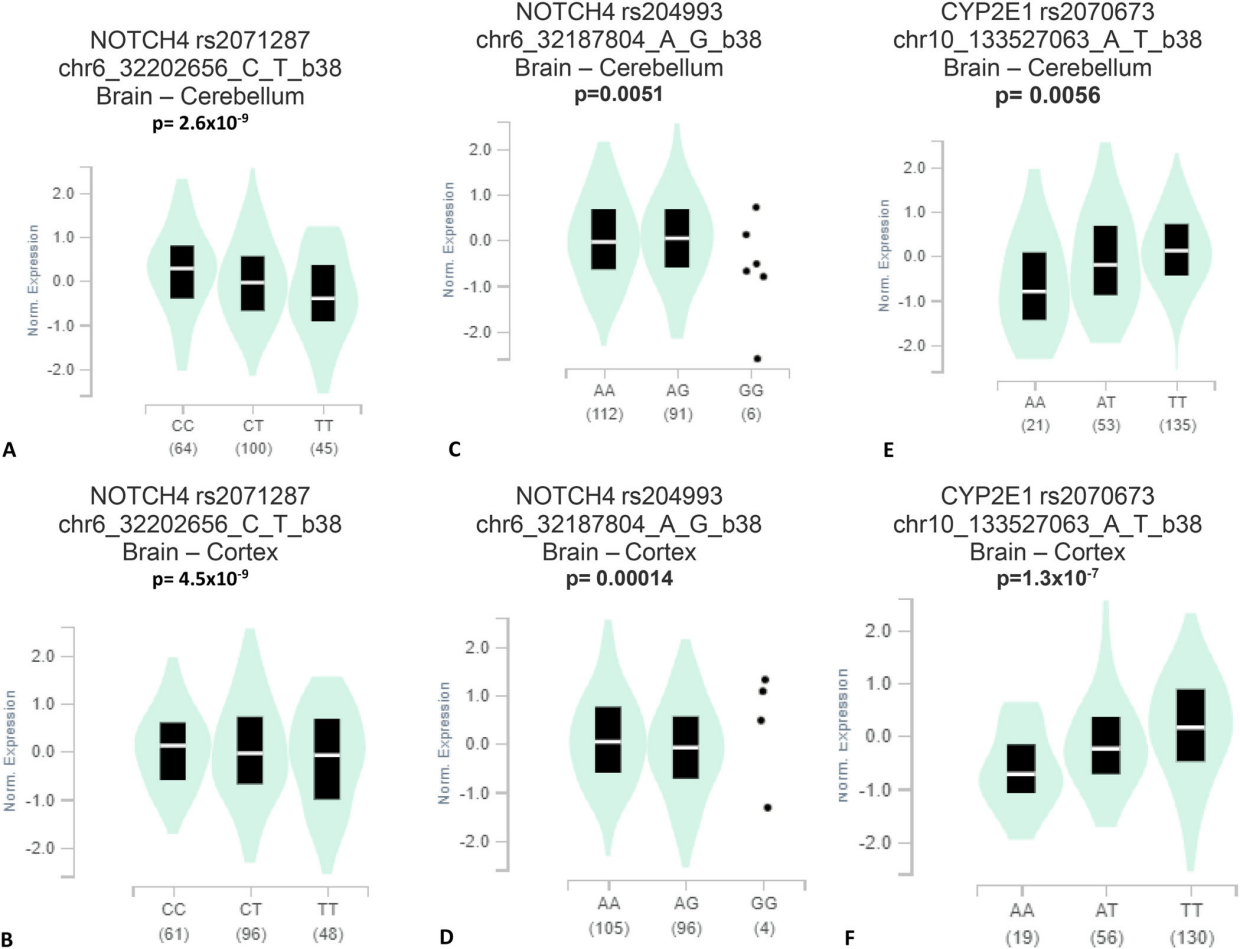
Genotype‐tissue expression (GTEx) of *NOTCH4* rs2071287polymorphism in (A) Brain – Cerebellum, (B) Brain – Cortex; *NOTCH4* rs204993 in (C) Brain – Cerebellum, (D) Brain – Cortex; *CYP2E1* rs2070673 in (E) Brain – Cerebellum, (F) Brain – Cortex indicate a significant difference in the expression between the mutant genotypes compared to the wild genotypes.

It was found that a significant difference in the mutant allele and wild allele in the expression for *NOTCH4* rs2071287, *NOTCH4* rs204993, and *CYP2E1* rs2070673 polymorphisms both in the brain cerebellum and cortex (Figure 1).

## 4 Discussion

Approximately 60%–85% of people with SCZ have a familial history of the disorder, making it one of the most debilitating and chronic brain disorders. Though multiple genetic studies shed light on genetic susceptibility as the etiology of SCZ, the exact pathophysiology of this disorder is still obscured. Alpha‐7 nicotinic receptor: *DISC1*, *GRM3*, *GRM7*, *NOTCH4*, *COMT*, *CYP2E1*, *NRG1*, *RGS4*, and *G72* are some of the best‐evaluated genes identified by genome‐wide epidemiological studies. Based on recent studies, a negative correlation was also identified between SCZ and mutations of several genes, such as dystrobrevin (*DTNBP1*) and neuregulin 1 [34, 35, 36, 37, 38].

Through the binding of NOTCH family receptors to their appropriate ligands, NOTCH4 signaling is an evolutionarily conserved intercellular signaling pathway that controls interactions between physically adjacent cells. The mature cell‐surface receptor is formed by the heterodimerization of two polypeptide chains obtained from the proteolytic processing of the encoded preproprotein in the trans‐Golgi network [39]. This receptor could be important for the development of the liver, kidneys, and arteries [40]. In addition, the differentiation, proliferation, and death of brain cells are all controlled by *NOTCH* through the process of creating and morphogenesis of organs [17]. Thus, the mutation of this gene may lead to brain‐related problems like SCZ. Though this present study found no significant linkage of the *NOTCH4* rs2071287 variant with SCZ risk; however, a strong relationship of *NOTCH4* rs204993 polymorphisms with increased SCZ risk was observed in four genetic models, including additive model 2 (OR = 3.39, *p* = 0.0001); dominant model (OR = 1.84, *p* = 0.0032); recessive model (OR = 2.67, *p* = 0.0005); and allelic model (OR = 1.75, *p* = 0.0001). Similarly, a case‐control study in the Chinese Han community revealed that *NOTCH4* rs2071287 variant was not associated with SCZ risk, but NOTCH4 rs204993 variant was correlated to the progression of SCZ [39, 41]. Another study conducted in 218 Taiwanese families also found the association of *NOTCH4* rs204993 polymorphism with the susceptibility of SCZ [27]. However, a previous study conducted in Japanese populations found a positive connection between *NOTCH4* rs2071287 polymorphisms and SCZ risk, which was validated by a previous GWAS investigation and also a follow‐up assessment [26, 42]. SCZ‐related inherited variability may also be present in people of different racial backgrounds, as the A allele frequency of the rs2071287 varied (0.38 for Chinese Han, 0.31 for Japanese, and 0.36 for Bulgarian). SCZ may be caused by the rs2071287 as well as rs3131296 SNPs, both of which showed a clear linking dysfunction with each other, indicating that the rs2071287 SNP must be fixed [18, 43]. In contrast, we found no significant linkage of NOTCH4 rs2071287 polymorphisms in the Bangladeshi population in any genetic inheritance models.

CYP2E1 gene is responsible for breaking down endogenous substrates like ethanol, acetone, and acetal, as well as exogenous substrates like nitrosamines, benzene, carbon tetrachloride, and ethylene glycol. This gene may be involved in a wide range of processes, including gluconeogenesis, hepatic cirrhosis, diabetes, and cancer, because of its numerous substrates [44]. However, the present study identified the association of *CYP2E1* genetic polymorphisms (*CYP2E1* rs2070673) with SCZ susceptibility in four genetic models. Though the genotypic association of this gene with increased SCZ risk was identified in only the overdominant model (OR = 1.49, *p* = 0.0408); however, a strong protective nature of this gene with SCZ risk was found in additive model 2 (OR = 0.39, *p* = 0.0013); recessive model (OR = 0.45, *p* = 0.0002), and allelic model (OR = 0.61, *p* = 0.0004). A previous study also reported an association between rs2070673 of *CYP2E1* with the risk of SCZ [29]. An investigation with 100 healthy participants from the Chinese population demonstrated that the allele percentages of the variant rs2070673 (333A > T) differ significantly from those of Japanese, Korean, African American, European–American, and other regions of the Chinese population [45]. Another study of 400 healthy human volunteers identified a link between *CYP2E1* polymorphisms and the Chinese Han population's susceptibility to SCZ [46]. For the first time, we can say with certainty that rs2070673 may be connected with an elevated susceptibility of SCZ because we observed a strong association in four genetic models we tested, including additive model 2, recessive, overdominant and the allele model.

In silico genotype‐based gene expression in the brain cerebellum and cortex tissues indicates that there might be a link between the studied polymorphisms and brain function, as we found a significantly different gene expression of the variant allele carrier genes compared to the wild allele carrier genes and we also found a connection of variant allele with SCZ. FPRP and BFDP tests also confirmed our findings.

Despite reporting a novel association between rs204993 and rs2070673 variants and the development of SCZ in Bangladeshi populations, our research has some limitations that must be noted. This may be due in part to the fact that the sample size of the population is comparatively small. Additional SNPs that have been linked to SCZ in other populations should also be noted. It will be easier to predict the genetic basis of Bangladeshi schizophrenic patients because now we know the hereditary fundamentals of Bangladeshi patients.

## 5 Conclusion

To sum up, our study provides new evidence of the link between *NOTCH4* rs204993 and *CYP2E1* rs2070673 genetic variations and the progression of SCZ in Bangladeshi inhabitants. In our research, *NOTCH4* rs2071287 was not linked to the susceptibility of SCZ. However, future explorative investigations may be necessary to confirm our findings.

## Author Contributions

**Md. Shalahuddin Millat:** formal analysis, investigation, project administration, writing–original draft, writing–review and editing. **Joysree Roy:** formal analysis, writing–original draft, writing–review and editing. **Md. Atikur Rahman:** investigation, writing–original draft, writing–review and editing. **Md. Abdul Aziz:** data curation, project administration, investigation, writing–review and editing. **Safiqul Islam:** investigation, validation, writing–original draft. **Md. Mazharul Islam Chowdhury:** formal analysis, writing–original draft, writing–review and editing, visualization. **Md Abdul Barek:** data curation, methodology, writing–original draft, writing–review and editing. **Md. Saddam Hussain:** methodology, writing–review and editing, writing–original draft, resources. **Mohammad Sarowar Uddin:** methodology, resources, visualization, writing–original draft. **Shafayet Ahmed Siddiqui:** software, validation, writing–original draft. **Mohammad Safiqul Islam:** conceptualization, data curation, project administration, formal analysis, software, resources, supervision, validation, writing–review and editing.

## Conflicts of Interest

The corresponding author, Prof. Mohammad Safiqul Islam, PhD, is a member of the editorial board, and he will not be involved in any stage of decision‐making for this manuscript. The other authors declare no conflicts of interest.

### Transparency Statement

The corresponding author affirms that this manuscript is an honest, accurate, and transparent account of the study being reported; that no important aspects of the study have been omitted.

## Data Availability

The data that support the findings of this study are available from the corresponding author upon reasonable request.
